# European thromboprophylaxis practice amongst paediatric cardiology units

**DOI:** 10.1007/s00431-025-06351-x

**Published:** 2025-08-04

**Authors:** Ciara Ryan, Michal Odermarsky, Daniel De Wolf, Sean T. Kelleher, Estelle Naumburg, Zdenka Reinhardt, Christoph Male, Wim Helbing, Damien Bonnet, Colin J. McMahon

**Affiliations:** 1 Department of Paediatric Cardiology, Children’s Health Ireland at Crumlin, Dublin 12, D12 N512 Ireland; 2 Department of Clinical Sciences Lund, Paediatric Cardiology, Lund University, Skane University Hospital, Lund, Sweden; 3 Paediatric and Congenital Cardiology, University Hospitals Gent and Brussels, Gent and Brussels, Belgium; 4 Institution of Clinical Science, Paediatrics, Umeå University, Umeå, Sweden; 5 Department of Paediatric Cardiology, Freeman Hospital, Newcastle, England; 6 Department of Paediatrics, Medical University of Vienna, Waehringer Guertel 18-20, Vienna, Austria; 7 Erasmus MC – Sophia Children’s Hospital, Dr. Molewaterplein 40, 3015 GD Rotterdam, The Netherlands; 8 Unité Médico-Chirurgicale de Cardiologie Congénitale Et Pédiatrique, Centre de Référence Malformations, Cardiaques Congénitales Complexes - M3C, Hôpital Necker Enfants Malades, APHP Université Paris Descartes, Sorbonne Paris Cité, 149 Rue de Sèvres, 75015 Paris, France; 9 School of Medicine, University College Dublin, Belfield, Dublin 4, Ireland

**Keywords:** Thromboprophylaxis, Anticoagulation, Aspirin, Warfarin, Bivalirudin, DOAC, Direct oral anticoagulants, Congenital heart disease, Paediatric

## Abstract

Children with congenital and acquired heart disease have an increased risk of thromboembolic events. There is a paucity of evidence on the optimal approach to thromboprophylaxis for this population, and the introduction of direct oral anticoagulants in recent years has led to significant variation in clinical practice. The aim of the study was to describe current practice in thromboprophylaxis prescribing amongst European Paediatric Cardiology units managing paediatric and congenital heart disease. A structured and approved online survey (SurveyMonkey.com) was developed for distribution to AEPC affiliated paediatric cardiology centres in Europe. Responses were received from 30 participants working in 27 Paediatric Cardiology centres across Europe. There is significant variation between participants in the approach to thromboprophylaxis in different patient cohorts, including functional single ventricle patients, post-cardiac catheterisation intervention/electrophysiology study, post-prosthetic valve insertion, cardiomyopathy/heart failure, and infective endocarditis. Differences were seen in the indications for thromboprophylaxis, as well as the agents used and the duration of treatment.

*Conclusion*: There is significant variation in practice amongst participants in Europe. Further studies are required in order to develop evidence-based guidelines for clinical practice.

**Table:** 

**What is Known:** • *Children with congenital and acquired heart disease have increased predisposition to thrombosis after specific surgeries and interventions.* • *International guidelines exist for the management of antiplatelet and anticoagulation agents in children with congenital and acquired heart disease.*
**What is New:** • *This study outlines the variation in practice amongst clinicians in Europe in the indications for thromboprophylaxis, medications used and duration of treatment for both congenital and acquired heart disease in paediatric patients.* • *This study highlights the need for up-to-date, evidence-based thromboprophylaxis guidelines for this patient population, including recommendations on direct oral anticoagulant use.*

**Supplementary Information:**

The online version contains supplementary material available at 10.1007/s00431-025-06351-x.

## Introduction

Paediatric cardiology and cardiothoracic surgery have evolved significantly over the past number of decades, with subsequent improvements seen in survival rates for children with significant heart disease [1]. However, paediatric and congenital heart disease continue to be associated with an increased risk of thromboembolic disease, with rates of thrombus detection ranging from 8 to 33%, depending on the underlying anatomy [2–4]. Thromboprophylaxis has become commonplace in paediatric cardiology patients. Numerous additional factors have to be considered in addition to pharmacological efficacy in paediatric patients. These include anxiety and needle phobia surrounding blood sampling and ease of dose titration based on available drug preparations. The burden of monitoring associated with vitamin K antagonists (VKAs) such as warfarin can be significant, and achieving a target therapeutic international normalised ratio (INR) range is particularly challenging in paediatric patients, with time in therapeutic range typically less than 70% [5]. Studies have shown that individual patient response to antiplatelet therapy is variable and has been demonstrated to be inadequate in over one third of patients undergoing high-risk cardiac procedures [6]. With this in mind, the therapeutic strategy needs to be carefully chosen in order to maximise the therapeutic benefit and minimise the burden on both the patient and their family.

Despite its frequent use in clinical practice, there is a paucity of evidence in relation to the optimal approach to thromboprophylaxis for these patients. This scarcity of evidence, coupled with the introduction of direct oral anticoagulants in recent years, has led to significant variation in clinical practice internationally. Current guidelines are outdated and are not reflective of the therapeutic strategies available to clinicians [2].

Different approaches to thromboprophylaxis exist both between and often within paediatric cardiology centres. The aim of the study was to describe current practice in thromboprophylaxis prescribing amongst paediatric cardiology units managing paediatric and congenital heart disease across Europe.

## Methods

A structured and approved online survey (SurveyMonkey.com) was developed and reviewed by eight consultant cardiologists, members of the Association for European Paediatric and Congenital Cardiology (AEPC) trials group committee. It was distributed to AEPC-affiliated consultant paediatric cardiologists practising in European centres, between September 2023 and February 2024. The survey consisted of 53 questions (see supplementary material) including open-ended and closed questions. The survey was distributed via email, and three reminder emails were sent to non-responders.

Current thromboprophylaxis practices in patients with congenital heart disease were surveyed. The treatment strategy used in specific patient cohorts was assessed, including functional single ventricle, post-insertion of prosthetic devices or surgical valve replacement, post-cardiac catheterisation or electrophysiology study, cardiomyopathy or heart failure, and infective endocarditis.

Data was collated on and statistical analysis was performed using Excel version 16. Ethical approval was waived by Children’s Health Ireland at Crumlin, Dublin, Ireland, as this was survey-based research.

## Results

**Fig. 1:**
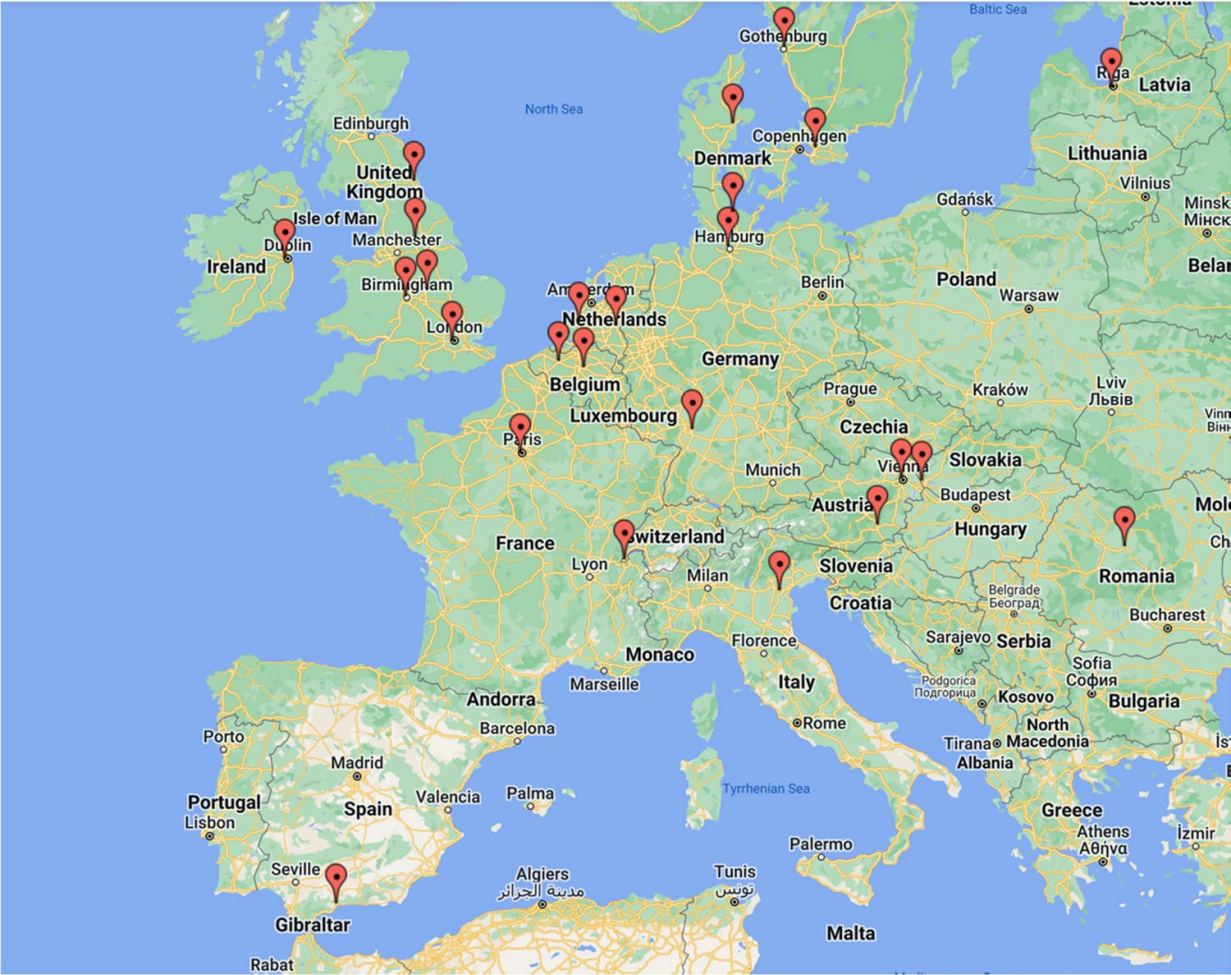
Map demonstrating participants’ location of practice across Europe

Responses were received from 30 paediatric cardiologists across Europe of 32 surveyed, giving a 93% response rate (Fig. 1).

### Anticoagulation guidelines

National guidelines for anticoagulation in CHD were reported to be present by ten participants (33.3%) with 22 participants (73.3%) reporting having local guidelines in their centre. Twenty-eight participants (93.3%) agreed it would be helpful to have clearer guidelines on optimal thromboprophylaxis use in specific congenital heart diseases.

### Thromboprophylactic agents

**Fig. 2:**
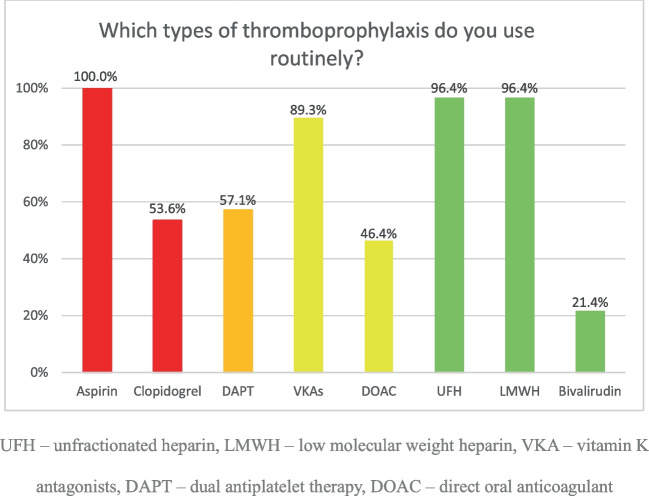
Thromboprophylaxis agents routinely used by participants. UFH, unfractionated heparin; LMWH, low molecular weight heparin; VKA, vitamin K antagonists; DAPT, dual antiplatelet therapy; DOAC, direct oral anticoagulant

Aspirin was the most commonly used agent, being prescribed by all 30 responding participants (100%) (Fig. 2).

### Fontan circulation

Following bidirectional Glenn procedure, 22 of 29 participants (75.9%) used routine thromboprophylaxis while seven participants (24.1%) did not.

Following Fontan completion, the most commonly used approach included an initial period of anticoagulation followed by transition to aspirin, which was used by nine participants (30%).

Thromboprophylaxis strategy was largely similar across different Fontan subtypes. See supplementary material for further details.

The reported duration of VKA use following Fontan completion varied from 3 months to lifelong therapy. Of the 28 participants that used VKA post-Fontan, the most frequently used approach was lifelong VKA therapy reported by ten participants (35.7%). One participant (3.5%) continued VKAs until adulthood at which point they employ a shared decision making pathway.

Long-term DOAC use in patients post-Fontan completion was reported by 11 participants (36.7%). Four participants (13.3%) commented that DOACs were either not available or not reimbursed for paediatric use in their country of practice.

When transitioning from anticoagulation to another agent post-Fontan, aspirin was the most commonly used long-term agent. Of the 24 participants who reported the agent they prescribed following initial anticoagulation, eight continued anticoagulation lifelong. Of the remaining 16 participants, 14 (87.5%) transitioned to aspirin monotherapy, one (6.3%) transitioned to either aspirin or clopidogrel monotherapy, and one (6.3%) transitioned to either aspirin or DAPT.

**Fig. 3:**
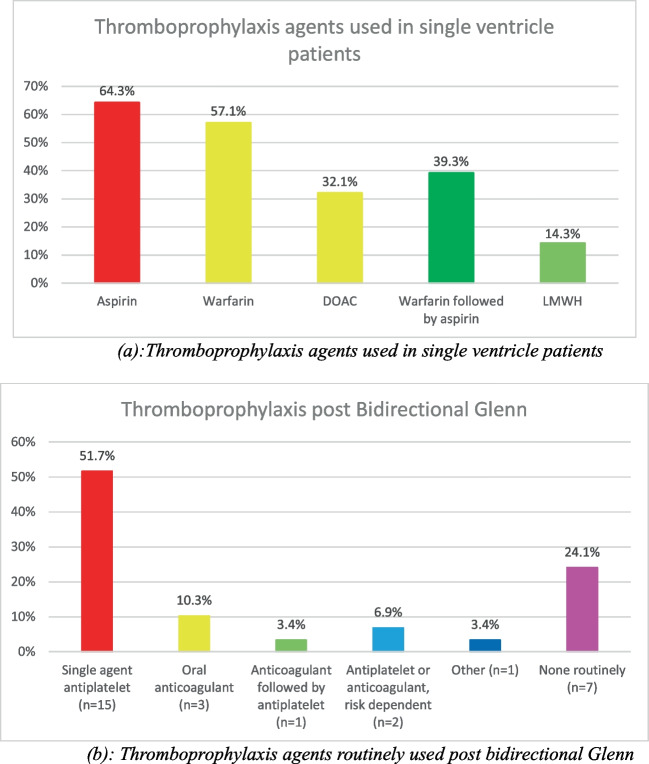

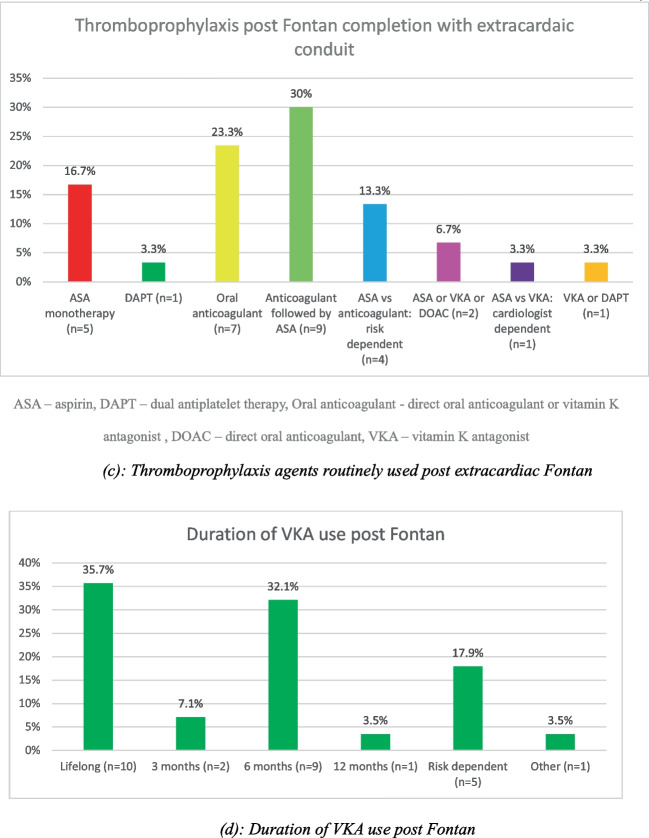
Thromboprophylaxis in single ventricle patients. **a** Thromboprophylaxis agents used in single ventricle patients. **b** Thromboprophylaxis agents routinely used post-bidirectional Glenn. **c** Thromboprophylaxis agents routinely used post-extracardiac Fontan. **d** Duration of VKA use post-Fontan. ASA, aspirin; DAPT, dual antiplatelet therapy; Oral anticoagulant, direct oral anticoagulant or vitamin K antagonist; DOAC, direct oral anticoagulant; VKA, vitamin K antagonist

In patients with failing Fontan circulation, 18 participants (62.1%) changed their thromboprophylaxis strategy. Many describe this decision being made on a case-by-case basis, depending on the patient’s systolic function, hepatic function, and history of thromboembolism. Adjusted treatment strategies reported include increasing target INR, changing from antiplatelet agents to VKAs, DOAC, or LMWH (Fig. 3).

### Thromboprophylaxis following interventional catheterisation (Fig. 4)

**Fig. 4:**
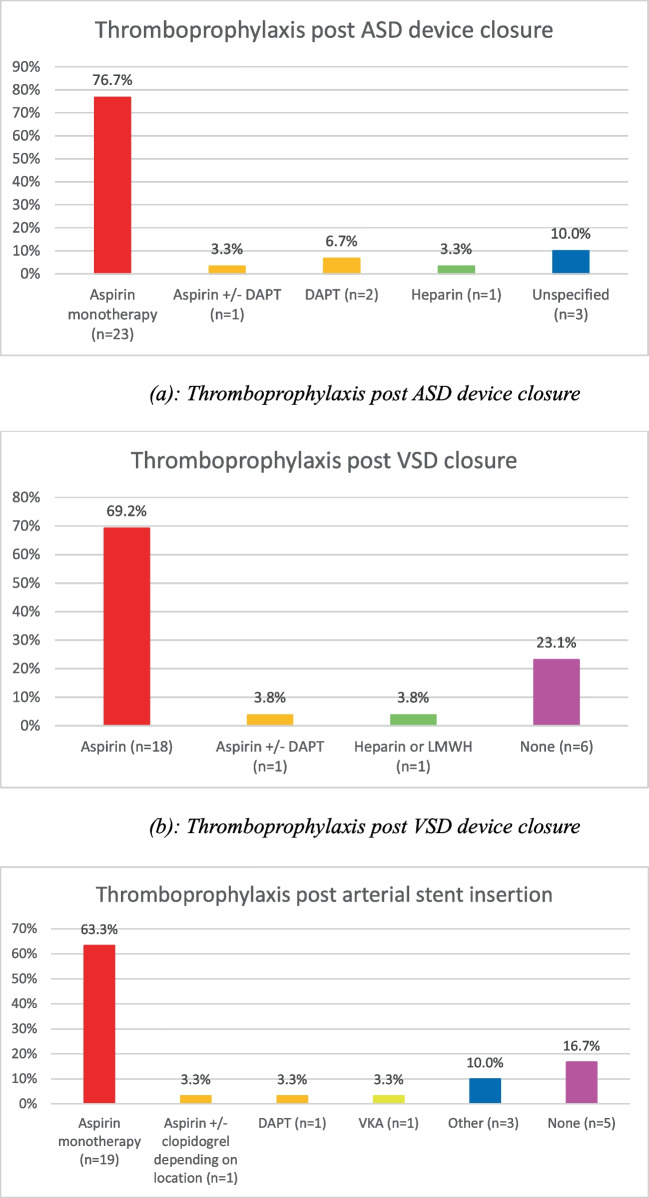
Thromboprophylaxis post-transcatheter device insertion. a Thromboprophylaxis post-ASD device closure. b Thromboprophylaxis post-VSD device closure. c Thromboprophylaxis post-arterial stent insertion

### Arterial catheterisation

During arterial catheterisation procedures, 23 participants (76.7%) gave prophylactic anticoagulation with unfractionated heparin bolus.

Following arterial catheterisation, 15 participants (50%) did not routinely continue thromboprophylaxis. The 15 participants who did included nine (30%) who used UFH, five (16.7%) who used LMWH, and one (3.3%) who used UFH in newborns but LMWH in all other cases.

In the case of loss of pulse post-arterial catheterisation, the majority of participants initiated treatment with UFH (*n* = 17, 58.6%). Less commonly adopted strategies included five participants (17.2%) who used UFH followed by a thrombolytic agent, three participants (10.3%) who used either UFH or LMWH, one participant (3.4%) who used LMWH, one participant (3.4%) who used thrombolytic therapy alone, one participant (3.4%) who used UFH in combination with vasodilator therapies, and one participant (3.4%) who used vasodilator agents alone.

### Radiofrequency ablation

Following radiofrequency ablation, 22 participants of 29 (75.9%) routinely prescribed thromboprophylaxis, five participants (17.2%) did not, and two participants (6.9%) considered it in certain cases. See supplementary material for further details.

### Valve replacement

a Bioprosthetic valves

**Fig. 5:**
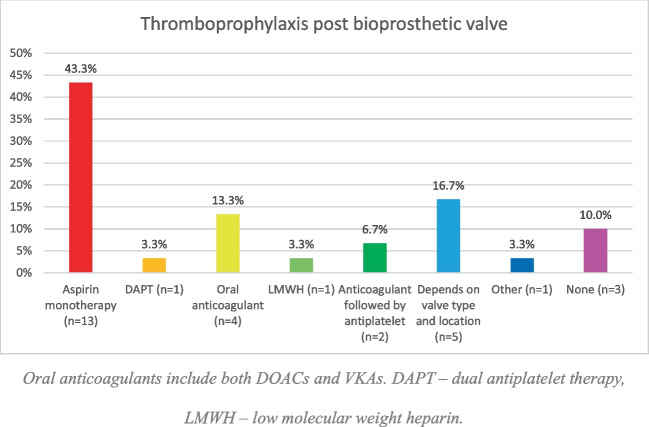
Thromboprophylaxis post-bioprosthetic valve insertion. Oral anticoagulants include both DOACs and VKAs. DAPT, dual antiplatelet therapy; LMWH, low molecular weight heparin

Almost all participants (90%) routinely prescribed thromboprophylaxis following bioprosthetic valve insertion, with only three participants (10%) not using any thromboprophylaxis (Fig. 5).

b Homografts

Similar results were seen for management of homografts, with thromboprophylaxis being used routinely by 22 participants (73.3%) and eight participants (26.7%) not using routine treatments. Aspirin monotherapy was used routinely by 19 participants (63.3%) for a duration ranging from 3 months to lifelong.

c Mechanical valves

Mechanical valves were managed by all participants with VKAs. Nineteen participants (63.3%) continued VKA therapy lifelong, with eight participants (26.7%) using VKAs for an unspecified duration of time and three participants (10%) choosing anticoagulation duration depending on valve type and position.

### Cardiomyopathy and heart failure

For stable outpatients with dilated cardiomyopathy (DCM), nine participants (30%) individualised patient treatment depending on ventricular function or individual risk profile. Thresholds reported for commencement of treatment included left ventricular ejection fraction (LVEF) < 25–30%, LVEF < 30%, and LVEF < 35%. Aspirin monotherapy was the commonest treatment regime used by 12 participants (40%).

In the event of decompensation with DCM requiring inotropic support, the most commonly used approach by 19 participants (63.3%) was to commence thromboprophylaxis with UFH or LMWH.

In the setting of left ventricular hypertrabeculation, 21 participants (70%) reported using thromboprophylaxis routinely, with six participants (20%) not. Four participants (13.2%) decided on treatment based on function, and one participant (3.3%) stated this is subject to debate.

For patients with arrhythmogenic right ventricular cardiomyopathy (ARVC) who develop arrhythmia, responses were notably heterogeneous, with a higher level of uncertainty reported by participants. Eleven participants (37.9%) routinely started thromboprophylaxis in this cohort, with further details available in supplementary material.

### Infective endocarditis (IE)

Most participants (42.9%) reported not giving routine thromboprophylaxis for IE.

See supplementary material for further details.

### Medication reimbursement

Thromboprophylaxis agents were reimbursed in the countries of 24 participants (82.8%).

See supplementary material for further details.

## Discussion

Paediatric patients with congenital and acquired heart disease represent a heterogenous group with complex and varied anatomy and physiology. Many of these children are at increased risk of thromboembolic events based on their underlying anatomy, with additional risk factors including the presence of prosthetic material, recent interventions, hepatic or haematological dysfunction, and impaired cardiac function.

Rates of thrombotic events range from 8 to 25% in patients post-Fontan [7, 8], and rates of intracardiac thrombus in patients with DCM are reported to be as high as 14% [4].

Despite this high level of morbidity associated with thrombotic events, there is significant variability in all aspects of thromboprophylaxis demonstrated in this survey from indications to commence treatment, choice of agent, as well as dose and duration of thromboprophylaxis.

The American Heart Association (AHA) guidelines on prevention and treatment of thrombosis in paediatric and congenital heart disease were developed over 10 years ago, and while they are very comprehensive, they are limited by the scarcity of comprehensive randomised controlled trials evaluating safety and efficacy of thromboprophylaxis agents in paediatric patients [2]. As such, recommendations are largely based on levels B and C evidence and often based on data from adult studies.

Discrepancies have been shown between current clinical practice and the American College of Chest Physicians (ACCP) guidelines for antithrombotic therapy for children and neonates [9]. A prospective audit carried out in a tertiary paediatric hospital showed that compliance with ACCP guidelines on thromboprophylaxis for all patients, including those with complex cardiac conditions, was as low as 50% [10]. Reasons proposed for this divergence from guideline recommendations include use of thromboprophylaxis in instances that are not currently addressed by clinical guidelines, or in areas in which evidence is insufficient to make recommendations in support of routine thromboprophylaxis.

### Single ventricle patients

The benefits of thromboprophylaxis in patients post-Glenn has not been well studied, and there is insufficient evidence to recommend routine thromboprophylaxis for all patients [11].

Single centre studies of long-term outcomes post-Fontan surgery report 10-year survival rates in excess of 95% in the modern surgical era, where the majority of patients undergo extracardiac conduit type Fontan procedures [12, 13]. Risk factors for thrombosis in this group include all three elements of Virchow’s triad, namely endothelial dysfunction, abnormal blood flow, and hypercoagulability [14]. Studies have also shown thromboembolic events are often clinically silent, and intracardiac thrombus may be undetectable on transthoracic echo, with reported intracardiac thrombus rates of up to 33% on transoesophageal echo [15].

Thromboprophylaxis is recommended for all patients with Fontan circulation because of the increased risk of thromboembolic events; however, the most effective strategy has not been demonstrated [16]. Multiple factors influence the therapeutic strategy, including time interval since Fontan completion, presence of a patent fenestration, history of thrombotic events, liver dysfunction, and Fontan failure. There remains significant variability in the approach to thromboprophylaxis amongst different physicians and different centres, as we demonstrated.

Observational studies have shown aspirin to be non-inferior to warfarin in terms of rates of cerebrovascular injury and thromboembolic events, with aspirin being superior in terms of bleeding risk [17–19]. Additionally, long-term warfarin use is associated with lower bone mineral density, potentially increasing the lifetime risk of fractures [18]. Despite this, our results demonstrate that VKAs remain one of the most commonly used thromboprophylactic agents for all Fontan subtypes, either as lifelong monotherapy or for the initial post-operative period prior to transitioning to antiplatelet therapy.

As DOACs have become more widely used in clinical practice for anticoagulation, they have also begun to replace VKA use in adult CHD populations in some countries, despite a lack of randomised clinical trials assessing their safety and efficacy [20, 21].

The UNIVERSE study was a pivotal trial, which compared rivaroxaban to aspirin for primary prophylaxis in paediatric single ventricle patients who had recently undergone Fontan completion [7]. Rates of bleeding events were similar between both groups, with a lower proportion of thrombotic events in the rivaroxaban group compared to the aspirin group (2% vs 9%). This was one of the first studies to examine DOAC use in paediatric CHD patients, and its findings have challenged the traditional approach to anticoagulation in this patient cohort, with many centres migrating towards rivaroxaban in place of VKAs [22]. Subsequent meta-analyses have further supported the use of DOACs in this population, showing their association with the greatest reduction in thromboembolic events, although a slightly increased risk of major bleeding when compared with aspirin and warfarin [23]. Other DOACs such as edoxaban and apixaban have also begun to be studied in paediatric patients with cardiac disease, and initial results have been promising, showing their potential as an alternative to current strategies [24, 25].

In one of the largest studies to date of apixaban use in children, Van der Pluym and colleagues showed apixaban is a safe alternative to standard therapy for the treatment and prevention of thrombosis in children with cardiac disease [26].

The benefits of DOACs include their standardised dosing regime, reduced food and drug interactions, and the lack of therapeutic level monitoring needed, making them an attractive alternative to current first-line paediatric therapies [27].

The safety and efficacy of DOACs in this cohort, as demonstrated by recent studies, should prompt their inclusion in clinical guidelines.

### Cardiac catheterisation

Anticoagulation during cardiac catheterisation was reported to be administered by the majority of participants, in line with AHA guidelines. Variation in anticoagulation in the cardiac catheterisation lab has been previously described including in monitoring of anticoagulation during catheterisation, protamine use, and outpatient anticoagulation after catheterisation [28]. This is thought to be contributed to by a combination of patient-specific factors and operator experience.

Thromboprophylaxis following cardiac catheter interventions was more standardised amongst participants, with aspirin being the mainstay of therapy following insertion of stent material or closure device. DAPT use was reported by a minority of participants, and evidence supporting its use in paediatric patients is limited. Adult studies have shown benefit of clopidogrel in addition to aspirin in coronary artery disease [29], and this data has been used in the development of guidelines for management of coronary artery disease in Kawasaki disease [30].

Management of loss of arterial pulse post-cardiac catheterisation was not uniform amongst participants. While AHA guidelines suggest initial treatment with UFH followed by transition to fibrinolytic therapy if limb ischaemia persists, a minority of centres described commencing treatment with LMWH either alone or in combination with vasodilator agents. Local variations in practice may relate to institutional guidelines, and practitioner responses may vary depending on the clinical scenario and examination findings present on a case-by-case basis.

### Valve replacement

Robust European and American adult guidelines exist for the management of valvular heart disease, including thromboprophylaxis following prosthetic insertion [31, 32]. Comparable paediatric guidelines are lacking due to a paucity of prospective, large-scale studies with long-term follow-up. Recommendations are largely based on low levels of evidence and are extrapolated from adult data [2]. Despite this, current practice is largely in line with current guidelines, acknowledging variations reported by single centres.

Adult guidelines for management of mechanical valves recommend that all patients with a mechanical valve are anticoagulated with a VKA [32]. The target INR recommended depends on the valve type and location, as well as individual risk factors for thrombosis including atrial fibrillation, previous thromboembolism, or left ventricular dysfunction. DOACs have not been adequately assessed for this indication and are therefore not recommended for patients with mechanical valves.

Following insertion of bioprosthetic valves, participants reported using a variety of thromboprophylaxis strategies, with aspirin being the most common. Management of mechanical valves was significantly more uniform, with all participants using VKAs and almost all continuing treatment lifelong.

### Cardiomyopathy and heart failure

The Recent European Society of Cardiology (ESC) guideline on management of cardiomyopathies refers to thromboprophylaxis as part of general management principles, in lieu of specific guidance on agents to use or threshold at which to commence treatment [33]. Recommendations on anti-coagulation are targeted towards adult patients with atrial arrhythmias in the setting of cardiomyopathy. The ESC guideline also notes the lack of data on long-term anticoagulation in children with DCM in sinus rhythm.

Data on the safety and efficacy of thromboprophylaxis in this cohort is limited, and existing guidelines give general recommendations largely targeted towards DCM or heart failure with reduced ejection fraction, as opposed to subtype specific guidance. Current international recommendations for commencing thromboprophylaxis include commencing VKAs no later than activation on transplant list [9], commencing either heparin or warfarin if there is intracardiac thrombus, if the patient has an ejection fraction less than 25% or fractional shortening less than 15% and a history of thromboembolism, or low ejection fraction and persistent uncontrolled atrial arrhythmia [34]. This contrasts with practice demonstrated by participants in our survey who reported higher thresholds of LVEF < 25–35% for commencement of thromboprophylaxis and more commonly used antiplatelet agents than anticoagulation.

A small-scale retrospective audit of 36 patients has shown the potential safety and efficacy of primary anticoagulation in severe cardiomyopathy [35]; however, more robust studies are required.

### Limitations

We acknowledge that the practice of individual participants may not necessarily be representative of the entire institution, and this survey did not examine variations in practice within institutions. Similarly, different centres within the same country may have varying approaches to thromboprophylaxis, and differences between centres in a single country were not explored in this study.

This survey did not explore the thromboprophylaxis practices for management of Kawasaki disease, deep venous thrombosis, or pulmonary embolism. This study did not explore in detail thromboprophylaxis dosing.

Given the survey nature of this study, limited information was available as to the clinical reasoning behind decision-making, and the rationale for different treatment strategies could not be explored in detail.

We note that local practice may be influenced by factors such as drug availability and financial reimbursement of medicines, which was not fully explored in this study.

## Conclusion

Thromboprophylaxis in paediatric and congenital heart disease is a challenging aspect of management in this heterogeneous patient population. There is a paucity of evidence-based clinical guidelines and significant variation in practice amongst clinicians. The optimal thromboprophylaxis strategy has not been clearly demonstrated for many conditions.

Emerging evidence suggests that DOACs may be a safe and effective alternative to current standard practice, which includes a combination of antiplatelet and VKA agents. This may reduce the burden on patients and families in relation to drug level monitoring, dietary restrictions, and frequent dose adjustments to maintain levels within a narrow therapeutic index. The development of evidence-based, paediatric-specific guidelines for thromboprophylaxis may assist clinicians in this challenging aspect of the management of congenital and acquired heart disease.

## Supplementary Information

Below is the link to the electronic supplementary material.

Supplementary file1 (DOCX 68 KB)

## Data Availability

No datasets were generated or analysed during the current study.

## Abbreviations

ACCP: American College of Chest Physicians
AEPC: Association for European Paediatric and Congenital Cardiology
ARVC: Arrhythmogenic right ventricular cardiomyopathy
ASA: Acetylsalicylic acid (aspirin)
ASD: Atrial septal defect
DAPT: Dual antiplatelet therapy
DCM: Dilated cardiomyopathy
DOAC: Direct oral anticoagulant
EF: Ejection fraction
ESC: European Society of Cardiology
INR: International normalised ratio
IU: International units
KG: Kilogramme
LMWH: Low molecular weight heparin
LVEF: Left ventricular ejection fraction
MM: Millimetre
UFH: Unfractionated heparin
VKA: Vitamin K antagonist
VSD: Ventricular septal defect
PDA: Patent ductus arteriosus
