# Supplementary material for: European thromboprophylaxis practice amongst paediatric cardiology units
Source: Eur J Pediatr. 2025 Aug 4;184(8):528. doi: 10.1007/s00431-025-06351-x (PMC12321674; doi:10.1007/s00431-025-06351-x)
Supplement: Supplementary file 1 — Supplementary file1 (DOCX 68 KB) [file 431_2025_6351_MOESM1_ESM.docx]

Supplementary Material

*Drug Monitoring*

VKAs were monitored by 29 participants (96.7%) using international normalised ratio (INR) levels. 24 participants (82.8%) used a combination of formal laboratory INR measurement through serum samples as well as point of care testing using systems such as CoaguChek (Roche Diagnostics), with just 3 participants (10.3%) using only formal laboratory INR levels, and 2 participants (6.9%) using only point of care testing systems.

Unfractionated heparin (UFH) was monitored by all 30 participants (100%) with 21 participants (70%) monitoring low molecular weight heparin (LMWH). DOAC monitoring was performed by only 2 participants (6.9%).

*Fontan Circulation*

ASA – aspirin, DAPT – dual antiplatelet therapy, DOAC – direct oral anticoagulant, VKA – vitamin K antagonist

*Figure 1: Thromboprophylaxis strategy by Fontan subtype*

Thromboprophylaxis strategy was largely similar across different Fontan subtypes. Lateral tunnel and classic Fontan subtypes were less frequently performed and 8 participants (27.5%) did not encounter these patients frequently enough to comment on the thromboprophylaxis regime used. One participant (3.7%) reported lateral tunnel being the only subtype of Fontan completion currently being performed. VKA monotherapy was used more often in those with classic Fontan compared to other subtypes.

*Thromboprophylaxis following interventional catheterisation*

Following patent ductus arteriosus (PDA) device closure, the majority of participants did not use thromboprophylaxis. Of 29 responses, 23 participants (79.3%) did not routinely use thromboprophylaxis, 1 participant (3.4%) used aspirin for some patients but not all, and 4 participants (13.8%) used aspirin for between 1 and 6 months.

Antiplatelet therapy was used by 21 participants (70%) following transcatheter insertion of arterial stents. 3 participants considered treatment on a case by case basis including 1 participant (3.3%) who used aspirin for pulmonary artery stents less than 5 mm in size, 1 participant (3.3%) who used thromboprophylaxis post pulmonary artery stenting in single ventricle patients, and 1 participant (3.3%) who used thromboprophylaxis post PDA stent insertion in patients with duct dependent pulmonary circulation.

The choice of stent dictated choice of thromboprophylaxis therapy for 7 participants (23.3%) with stent size and location being reported to be influencing factors.

*Figure 2: Thromboprophylaxis post arterial stent insertion*

*Prosthetic valves*

*Figure 3: Thromboprophylaxis post bioprosthetic valve insertion*

*Figure 4: Thromboprophylaxis post homograft insertion*

*Cardiomyopathy*

*Figure 5: Thromboprophylaxis in outpatients with Dilated Cardiomyopathy*

*Figure 6: Thromboprophylaxis in decompensated with Dilated Cardiomyopathy requiring inotropic agents*

*Figure 7: Thromboprophylaxis in patients with left ventricular hypertrabeculation*

2 participants (6.6%) decided between antiplatelet agent and anticoagulation depending on function, 2 participants (6.6%) based treatment initiation on function.

1 participant (3.3%) did not comment on treatments used, stating this is subject to debate.

*Figure 8: Thromboprophylaxis in patients with Arrhythmogenic Right Ventricular Cardiomyopathy*

11 participants (37.9%) routinely started thromboprophylaxis in this cohort with 7 participants (24.1%) being unsure or unable to comment, 5 participants (17.2%) decided on treatment on a case by case basis and a further 5 participants (17.2%) did not routinely start thromboprophylaxis. 2 participants (6.9%) used LMWH, one of whom only commenced treatment if the ejection fraction (EF) was less than 25%.

*Radiofrequency Ablation*

Following radiofrequency ablation, 22 participants (75.9%) routinely prescribed thromboprophylaxis, 5 participants (17.2%) did not, and 2 participants (6.9%) considered it in certain cases.

Aspirin was the most commonly used agent, being prescribed routinely by 16 participants (55.2%) and considered by 1 participant (3.4%) following left sided ablations. 1 participant (3.4%) used initial LMWH followed by aspirin for left sided ablations and 1 participant (3.4%) used initial UFH followed by aspirin for 3 months if trans-septal puncture was performed. The type of arrhythmia being treated determined the thromboprophylaxis for 1 participant (3.4%) who used aspirin post atrial flutter ablations and lifelong anticoagulation post atrial fibrillation ablations.

*Figure 9: Thromboprophylaxis post radiofrequency ablation*

*Infective Endocarditis (IE)*

Most participants (42.9%) reported not giving routine thromboprophylaxis for IE.

2 participants (7.1%) used aspirin routinely, with 1 participant (3.6%) considering aspirin only if left sided vegetations were present and 1 participant (3.6%) using either aspirin or no thromboprophylaxis. 1 participant (3.5%) used either UFH or aspirin depending on the case. 1 participant (3.6%) reported avoiding DOACs but did not specify which agent they routinely use.

*Figure 10: Thromboprophylaxis in patients with endocarditis*

*Medication Reimbursement*

Thromboprophylaxis agents were reimbursed in the countries of 24 participants (82.8%). Of the 18 participants who provided further details in relation to the specific medications which were reimbursed, 10 (55.6%) reported that all medicines can be reimbursed and 3 participants (16.7%) reported that all medicines except DOACs can be reimbursed. 2 participants (11.1%) reported only VKAs are reimbursed, 1 participant (5.5%) reported aspirin and VKAs are reimbursed, 1 participant (5.5%) reported aspirin, heparin, VKAs and DOACs are reimbursed and 1 participant (5.5%) reported 90% of medicines are reimbursed
